# Supplementary material for: DMRforPairs: identifying Differentially Methylated Regions between unique samples using array based methylation profiles
Source: BMC Bioinformatics. 2014 May 15;15:141. doi: 10.1186/1471-2105-15-141 (PMC4046028; doi:10.1186/1471-2105-15-141)
Supplement: Additional file 2 — DMRforPairs output for the comparison of A431-MCF7 and NA17018-NA17105. Please start from the HTML files in each folder. Available via the BMC Bioinformatics website. [file 1471-2105-15-141-S2.zip › 1394847754114233_MOESM2_ESM/A431_MCF7/figures/39318.pdf]

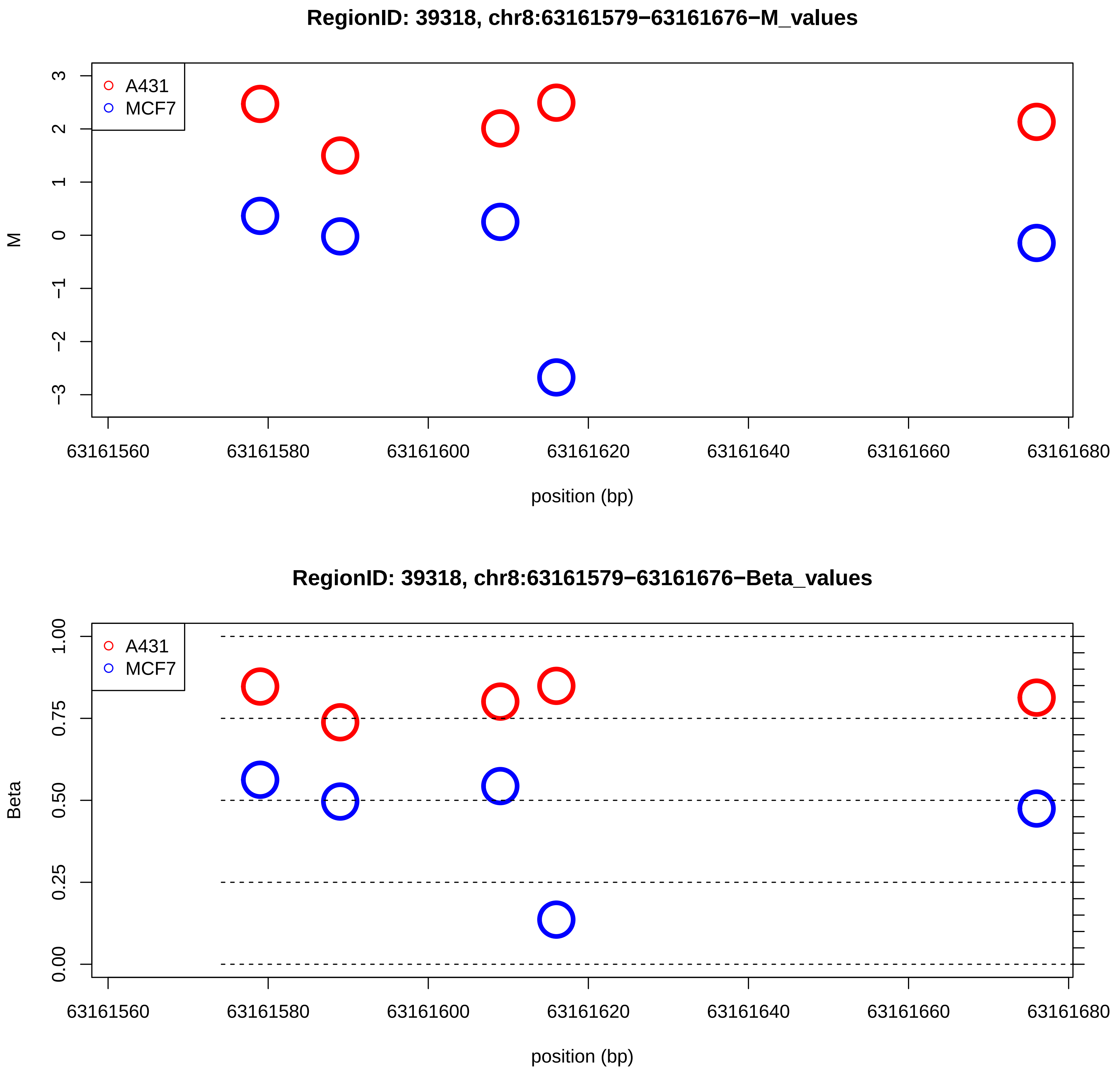

RegionID: 39318, chr8:63161579−63161676−M_values
A431
MCF7
M
position (bp)
63161560
63161580
63161600
63161620
63161640
63161660
63161680
RegionID: 39318, chr8:63161579−63161676−Beta_values
Beta

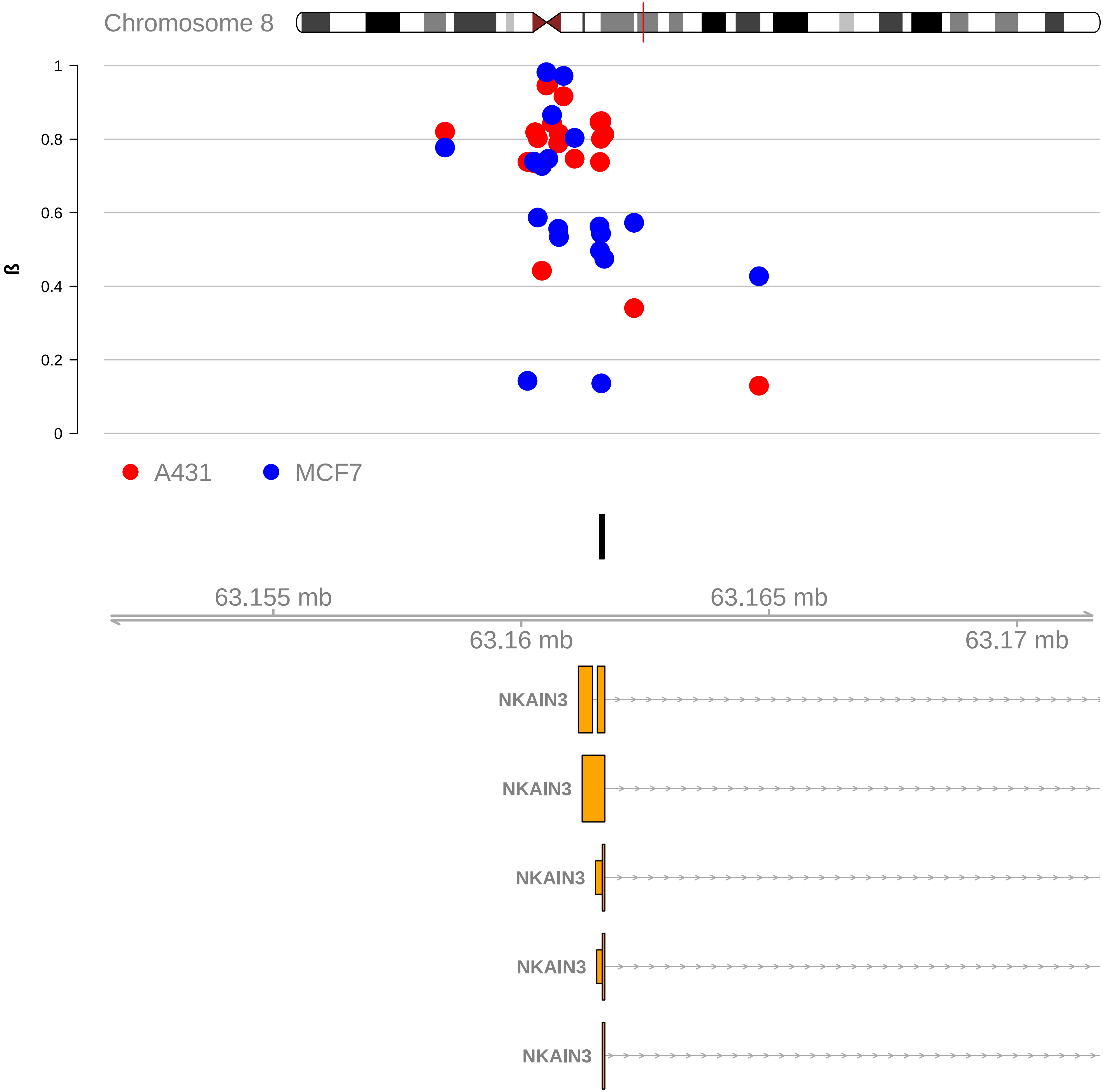

Chromosome 8
ß
1
0.8
0.6
0.4
0.2
0
A431
MCF7
63.155 mb
63.165 mb
63.16 mb
63.17 mb
NKAIN3
NKAIN3
NKAIN3
NKAIN3
NKAIN3
